# Genomic Profiling and Functional Analysis of let-7c miRNA-mRNA Interactions Identify *SOX13* to Be Involved in Invasion and Progression of Pancreatic Cancer

**DOI:** 10.1155/2020/2951921

**Published:** 2020-12-24

**Authors:** Shannon R. Nelson, Sandra Roche, Maura Cotter, Pablo Anton Garcia, Daniela Reitmeier, Elisabeth Zollbrecht, Fiona O'Neill, Martin Clynes, Padraig Doolan, Jai P. Mehta, Niall Swan, AnneMarie Larkin, Naomi Walsh

**Affiliations:** ^1^National Institute for Cellular Biotechnology, School of Biotechnology, Dublin City University, Dublin 9, Ireland; ^2^Histopathology Department, St. Vincent's University Hospital, Elm Park, Dublin 4, Ireland

## Abstract

**Background:**

Pancreatic cancer is a devastating disease; its lethality is related to rapid growth and tendency to invade adjacent organs and metastasize at an early stage.

**Objective:**

The aim of this study was to identify miRNAs and their gene targets involved in the invasive phenotype in pancreatic cancer to better understand the biological behaviour and the rapid progression of this disease.

**Methods:**

miRNA profiling was performed in isogenic matched high invasive and low-invasive subclones derived from the MiaPaCa-2 cell line and validated in a panel of pancreatic cancer cell lines, tumour, and normal pancreas. Online miRNA target prediction algorithms and gene expression arrays were used to predict the target genes of the differentially expressed miRNAs. miRNAs and potential target genes were subjected to overexpression and knockdown approaches and downstream functional assays to determine their pathological role in pancreatic cancer.

**Results:**

Differential expression analysis revealed 10 significantly dysregulated miRNAs associated with invasive capacity (Student's *t*-tests; *P* value <0.05; fold change = ±2). The expression of top upregulated miR-135b and downregulated let-7c miRNAs correlated with the invasive abilities of eight pancreatic cancer cell lines and displayed differential expression in pancreatic cancer and adjacent normal tissue specimens. Ectopic overexpression of let-7c decreased proliferation, invasion, and colony formation. Integrated analysis of miRNA-mRNA using *in silico* algorithms and experimental validation databases identified four putative gene targets of let-7c. One of these targets, *SOX13*, was found to be upregulated in PDAC tumour compared with normal tissue in TCGA and an independent data set by qPCR and immunohistochemistry. RNAi knockdown of *SOX13* reduced the invasion and colony formation ability of pancreatic cancer cells.

**Conclusion:**

The identification of key miRNA-mRNA gene interactions and networks provide potential diagnostic and therapeutic strategies for better treatment options for pancreatic cancer patients.

## 1. Introduction

Pancreatic cancer is fast becoming the major cause of cancer‐related mortality worldwide [1, 2]. With a rapid progression and spread, the 5-year survival rate of pancreatic cancer is 9%, and despite improvements in therapeutic technologies, the clinical outcome has not significantly improved in decades. As pancreatic cancer is notoriously asymptomatic and difficult to detect at an early stage, the majority of patients (approximately 80%) are diagnosed after the cancer has metastasized, making them ineligible for surgical resection, which is the only curative treatment [3]. However, even after resection, 85% of patients experience recurrence of the disease [4, 5]. Therefore, further understanding of the pathogenesis of pancreatic cancer can advance potential diagnostic and therapeutic strategies for the early detection and spread of pancreatic cancer.

MicroRNAs (miRNAs) are a family of ∼22 nucleotide endogenous, noncoding RNAs that can influence messenger RNA (mRNA) stability and translation. A single miRNA can interact with multiple target genes exerting oncogenic or tumour suppressive functions and can potentially regulate multiple cellular pathways. miRNA regulation of gene expression has been reported to be involved in many tumorigenic processes, including cell proliferation, migration, invasion, and metastasis [6]. Various miRNA expression profiling studies have identified specific miRNAs found to be associated with key phenotypic, clinical, and pathological characteristics of pancreatic cancer. Zhao et al. [7] reported that downregulation of miRNA-141 targets the tumour-promoting gene *MAP4K4* in *in vitro* and *in vivo* pancreatic cancer models. Previous studies have identified single miRNAs such as miR-323-3p [8], miR-224 [9], and miR-720 [10] as potential targets against pancreatic cancer cell migration and invasion. Members of the let-7 family have a highly conserved sequence and function from across species [11] and are critical regulators of many normal processes such as embryonic development, stem cell maintenance, differentiation, and glucose metabolism [12]. Multiple studies suggest that members of the let-7 family function as tumour suppressors in various cancers including pancreatic cancer [13]. Validated targets of let-7 deregulated in pancreatic cancer include *KRAS, STAT3*, *IGF2BP*, and *HMGA1*/*HMGA2* [12, 14, 15].

These studies highlight the important roles of miRNA in pancreatic cancer, and although a number of studies have identified miRNAs in pancreatic cancer progression, few analyses have been performed allowing for the identification of key miRNA-mRNA interaction networks involved in the progression and invasion of PDAC. Therefore, we hypothesized that by interrogating miRNA expression data from isogenic matched high-invasive and low-invasive subclones of a pancreatic cancer cell line, we would reveal crucial miRNAs involved in the invasion and progression of PDAC. Our analyses identified let-7c as significantly downregulated miRNA in invasive PDAC cells. Using *in silico* and experimental data, we identified the *SOX13* gene as a potential gene target. RNAi knockdown of *SOX13* reduced the invasion and colony formation ability of pancreatic cancer cells. These findings provide new insights into the mechanisms of the invasive phenotype of pancreatic cancer and may help to better understand the biological behaviour and the rapid progression of this cancer.

## 2. Materials and Methods

### 2.1. Tissue Culture of Cell Line Reagents

The human pancreatic cell lines Panc-1, SW1990, Capan-2, MiaPaCa-2 (ATCC, USA), HPAC (DSMZ, Germany), and BxPc-3 (ECACC, UK) were used in this study. Clone #3 and Clone #8 were previously isolated by single-cell dilution from MiaPaCa-2 in our laboratory [16]. Cells were maintained in a humidified atmosphere containing 5% CO_2_ at 37°C in Dulbecco's modified Eagles medium (DMEM) supplemented with 5% (*v*/*v*) foetal bovine serum and 2% *v*/*v* L-glut (Sigma–Aldrich). All cell lines were free from *Mycoplasma* as tested with the indirect Hoechst staining method. Authentication of human pancreatic cancer cell lines was confirmed by STR DNA fingerprinting.

### 2.2. miRNA Expression Profiling

TaqMan Low-Density miRNA Arrays (TLDA) of 384 human miRNAs were performed on miRNA isolated from high-invasive Clone #3 and low-invasive Clone #8, in three biological repeat experiments. miRNAs were isolated using the mirVana miRNA isolation kit (Applied Biosystems), following the manufacturer's instructions. RNA quantity and purity were assessed spectrophotometrically (NanoDrop ND-1000; Labtech International, Ringmer, East Sussex, UK). cDNA was synthesized using the TaqMan miRNA reverse transcription kit (Applied Biosystems) and Multiplex RT Human Primer Pool Sets. Resulting cDNA was then loaded onto TLDA cards according to manufacturer's instructions. TLDA cards were run on an ABI 7900HT Real Time PCR system (Applied Biosystems). The Ct values were obtained using the SDS v2.2 software with automatic threshold settings. All data were normalized to the geometric mean of endogenous controls RNU44 and RNU48. Following data normalization, miRNA lists were generated using a *t*-test to identify miRNAs that were significantly differentially expressed (*P* value <0.05; fold change = ±2) between high-invasive Clone #3 and low-invasive Clone #8. For each miRNA, the relative expression level was determined by the 2^−ΔΔCt^ value calculation formula.

### 2.3. qRT-PCR Analysis of miRNA Expression

The TaqMan miRNA Reverse Transcription Kit (Applied Biosystems, 4366597) was used to synthesize miRNA from total RNA. The RT-PCR was performed using a G-Storm thermocycler (Model GS1; Somerton Biotechnology Centre, Somerset, UK). Synthesized miRNA was stored at −20°C. Quantitative qPCR was used to assess miRNA expression (Applied Biosystems, 4453320) using the Applied Biosystems 7900 Real-Time PCR System. The relative miRNA expression was calculated using the equation 2^−ΔCt^, where ΔCt = Ct (miRNA) − Ct (RNU44/RNU48).

### 2.4. mRNA Expression Profiling

Microarrays using GeneChip Human Genome U133 Plus 2.0 Arrays (Affymetrix) were performed on RNA isolated from Clone #3 and Clone #8, in three biological repeat experiments. Total RNA was isolated using the RNeasy kit (Qiagen), following the manufacturer's instructions. RNA quantity and purity were assessed spectrophotometrically (NanoDrop ND-1000). The Agilent bioanalyzer was used to assess RNA qualitatively after isolation, after biotin-labelling and postfragmentation. Double-stranded cDNA was synthesized from 10 *μ*g total RNA using SuperScript II RT (Invitrogen Life Technologies) and T7-Oligo(dT)24 promoter primer (Affymetrix, Mercury Park, High Wycombe, UK) to initiate reverse transcription of mRNA. Following clean-up of double-stranded cDNA using the GeneChip Sample Cleanup Module (Affymetrix), biotin-labelled cRNA was synthesized using the Enzo Bioarray HighYield RNA Transcription Labelling Kit (Affymetrix), purified using IVT cRNA Cleanup Columns (Affymetrix), and fragmented to products of 35–200 bases.

Hybridization solution (1 mol/L NaCl, 20 mmol/L EDTA, 100 mmol/L 2-(N-morpholino)ethanesulphonic acid, and 0.01% Tween 20) was used to prehybridize Affymetrix U133 plus 2.0 microarrays for 10 min at 45°C. The prehybridization solution was removed and replaced with 200 *μ*L hybridization solution containing 0.05 *μ*g/*μ*L fragmented cRNA. The arrays were hybridized for 16 h at 45°C. Arrays were subsequently washed (Affymetrix Fluidics Station 400) and stained with streptavidin-phycoerythrin (Stain Buffer, 2 mg/mL acetylated BSA, and 10 *μ*g/mL streptavidin R-phycoerythrin; Molecular Probes, Inc., Eugene, OR, USA) and were scanned on a 2500 GeneArray scanner. Resulting data were analyzed using Affymetrix Transcriptome Analysis Console (TAC) software. Gene-level normalization and signal summarization were performed on all the data from the biological triplicates, assessed using the model-based probe logarithmic intensity error algorithm. Following data normalization, gene lists were generated using a *t*-test to identify genes that were significantly (*P* < 0.05, fold change = ±2) differentially expressed between the high-invasive Clone #3 arrays and the low-invasive Clone #8 arrays.

### 2.5. Cell Transient Transfection

All transfections were carried out using Lipofectamine 2000 (Invitrogen, #11668027). For let-7c miRNA overexpression functional experiments, pre-miR-let-7c (Ambion Inc, PM10436) and appropriate controls—pre-miR miRNA precursor (Ambion Inc, AM17100) and untreated negative controls—were used in each experiment. For target gene knockdown functional experiments, predesigned pool of 3 target-specific 19–25 nt siRNAs designed to knock down *SOX13* gene expression (Santa Cruz Biotech., sc-38424), a control (nontransfected), and a scrambled control siRNA (sc-37007) were used. For functional experiments, Panc-1 cells were seeded at a density of 3 × 10^5^ cells/mL in a 6-well plate with 30 nM pre-miR/siRNA. Transfection medium was removed after 24 h and replaced with fresh growth medium. The transfected cells were collected at 72 and 96 h for immunoblotting and assayed for changes in invasion, colony formation, and proliferation capacity at 72 h.

### 2.6. Invasion Assays

Invasion assays were performed as previously described [17]. Briefly, Matrigel (Sigma) was diluted to 1 mg/mL in serum-free DMEM. 100 *μ*L of diluted Matrigel was coated on each Boyden chamber insert (Falcon) (8.0 *μ*m pore size), in a 24-well plate (Costar) and incubated overnight at 4°C. The Matrigel was allowed to polymerize at 37°C for 1 h and then washed with DMEM. 100 *μ*L of complete DMEM was added to the wells, and 1 × 10^5^ cells/100 *μ*L of transfected and control cells were then seeded onto inserts. An aliquot of 500 *μ*L of complete DMEM was added into the underside of the well. After a 24-h incubation, the inside of the insert was wiped with a wet cotton swab. The under surface was gently rinsed with PBS and stained with 0.25% (*w*/*v*) crystal violet for 10 min, rinsed with sterile water, and allowed to dry. The inserts were then viewed under the microscope, and the number of cells per field in 10 random fields was counted at 200x magnification. The average number of cells per field was then multiplied by a factor of 140 (growth area of membrane/field area viewed at 200x magnification (calibrated using a microscope graticule) to determine the total number of invading cells. Experiments were performed in triplicate.

### 2.7. Cell Proliferation Assays

To observe the effects of miRNA let-7c or *SOX13* on proliferation, 72 h after transfection the highly invasive Panc-1 cells were harvested by trypsinization. Cell suspensions containing 1 × 10^4^ cells/mL were prepared in a cell culture medium. 100 *μ*L/well of the cell suspension and 100 *μ*L/well of media were added to 96-well plates (Costar, 3599). Plates were agitated gently in order to ensure even dispersion of cells over the surface of the wells. Cells were then incubated for a further 6-7 days until the control wells had reached approximately 80–90% confluency. Assessment of cell survival was determined by the acid phosphatase assay. Experiments were performed in triplicate. Results are graphed as percentage survival relative to the scrambled control cells.

### 2.8. Colony Formation Assays

Transfected and control cells were seeded in a 6-well plate at a concentration of 1 × 10^3^ cells/well in 2 mL of complete media. Media were changed every 3-4 days. After 10–14 days of incubation, cells were washed with PBS, then stained with 0.25% crystal violet for 10 min, and washed using water. Plates were allowed to dry, wells were then scanned, and the colonies were counted using Clono-counter [18]. Experiments were performed in triplicate. Results are graphed as percentage colony formation ability relative to the scrambled control cells.

### 2.9. Immunohistochemistry

4-*µ*m Formalin-fixed paraffin-embedded pancreatic tumour sections of tissue blocks were cut using a microtome, mounted onto poly-l-lysine-coated slides, and dried overnight at 37°C. Slides were stored at room temperature until required. Briefly, the slides were immunohistochemically stained using the primary antibody specific for *SOX13* (Abcam ab198921). After deparaffinization and rehydration, the slides were subjected to an antigen retrieval step consisting of 20-min incubation in pH 9.0 buffer (Target Retrieval Dako) in a 95°C water bath followed by cooling to room temperature. Staining was performed using an automated staining apparatus for IHC (Autostainer; Dako) according to the manufacturer's guidelines. Positive and negative controls were processed at the same time. The slides were counterstained with haematoxylin, dehydrated in graded alcohols, and mounted (DPX; Sigma). Scoring and interpretation were determined based on the intensity of staining in tumour using a semiquantitative scoring system. The intensity score was assigned as follows: 0 (negative), 1 (weak), 2 (moderate), and 3 (strong) by a pathologist (MC).

### 2.10. Computational Target Prediction *In Silico* Analysis

For the significantly dysregulated miRNAs in our data set, target prediction was carried out using PicTar [19] (https://pictar.mdc-berlin.de/cgi-bin/PicTar_vertebrate.cgi), TargetScan v7.2 [20] (http://www.targetscan.org/vert_72/), and miRDB [21] (http://mirdb.org/). The miRNA-mRNA interaction pairs predicted by 2 or more algorithms and supported with experimental evidence miRWalk v3.0 [22] (http://mirwalk.umm.uni-heidelberg.de/) and TarBase v8 [23] (http://carolina.imis.athena-innovation.gr/diana_tools/web/index.php?r=tarbasev8%2Findex) were considered as target relationship pairs.

### 2.11. Statistical Analysis

Student's *t*-tests (two-tailed, two-sample unequal variance) were used to analyze differences between comparisons. A *P* value <0.05 was considered statistically significant. Linear regression was performed to analyze the correlation between miRNAs and invasion abilities of the PDAC cell line panel. Kaplan–Meier analysis was performed on patients dichotomized by high and low *SOX13* expression based on the median expression levels, and statistical differences in the survival time between groups were compared using the log-rank test. One-way ANOVA was used in GEPIA to test the difference between normal and cancer samples. The statistical analyses were conducted using the STATA software package v13 and Graph Pad Prism v8.

## 3. Results

### 3.1. MicroRNA Expression Profiling of the High-Invasive and Low-Invasive Subclonal Populations

**Figure 1:**
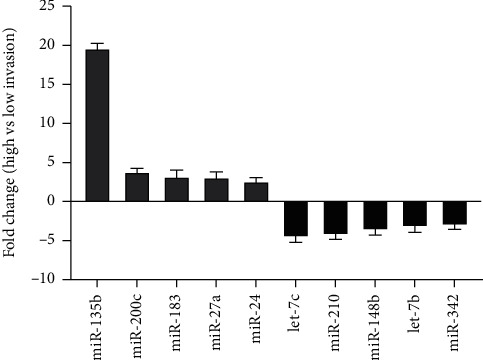
Significant differentially expressed microRNA between high- and low-invasive subclones of the pancreatic cancer cell line MiaPaCa-2. Data normalized to geometric mean of internal endogenous controls RNU44 and RNU48. Data displayed as RQ (2^−ΔΔCt^) fold change relative to high invasion (calibrator) *n* = 3.

TaqMan Human miRNA Low-Density Array (TLDA) analysis was performed to identify candidate miRNAs exhibiting altered expression between high-invasive versus low-invasive pancreatic cancer clonal cell lines, previously created in our lab [16]. After filtering for low-abundant miRNAs (Ct value > 37 and nondetectable), a set of 240 miRNAs (from total 365) remained for further analysis. The TLDA data were analyzed using the geometrical mean of endogenous controls RNU48 and RNU44 as reference genes to normalize qRT-PCR data (relative quantification 2^−ΔΔCt^). Comparative analysis of high vs. low invasion identified 10 differentially expressed miRNAs, five of which were upregulated and five were downregulated (*P* value <0.05 and fold change = ±2) (Figure 1). Among these, two miRNAs (let-7c and miR-135b) showed the highest statistical significance and fold change values and were selected for further validation.

### 3.2. Quantitative miRNA Validation with qRT-PCR

**Figure 2:**
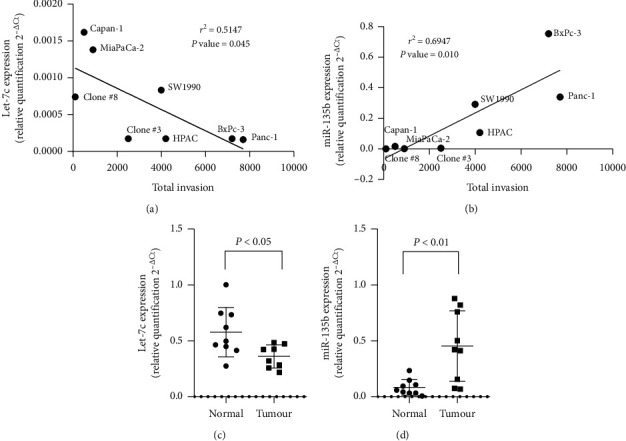
Expression of (a) let-7c and (b) miR-135b miRNA in eight pancreatic cancer cell lines correlated with invasion abilities. Expression of (c) let-7c and (d) miR-135b miRNA in adjacent normal pancreas tissue and PDAC tumour. Data are expressed as relative quantification 2^−ΔCT^ relative to geometric mean of endogenous controls RNU44 and RNU48.

The top underexpressing let-7c and overexpressing miR-135b miRNAs were quantified using qRT-PCR in eight established pancreatic cancer cell lines correlated with invasive abilities [17] and further quantified in PDAC tumour and adjacent normal pancreas tissues. An inverse linear correlation was observed between increased expression of let-7c and decreased invasive abilities in the eight cell lines (*r*^2^ = 0.5147, *P* value = 0.045), whereas an increasing correlation of miR-135b expression in the more invasive cell lines such as Panc-1 and BxPc-3 (*r*^2^ = 0.6947, *P* value = 0.010) was observed (Figures 2(a) and 2(b)). Significant increased expression of let-7c was detected in normal adjacent pancreas tissue compared with tumour (*P* value <0.05), whereas miR-135b was significantly decreased in adjacent normal pancreas tissue compared with tumour (*P* value <0.01) (Figures 2(c) and 2(d)).

### 3.3. let-7c Overexpression Inhibited Growth, Invasion, and Colony Formation of Pancreatic Cancer Cells *In Vitro*

**Figure 3:**
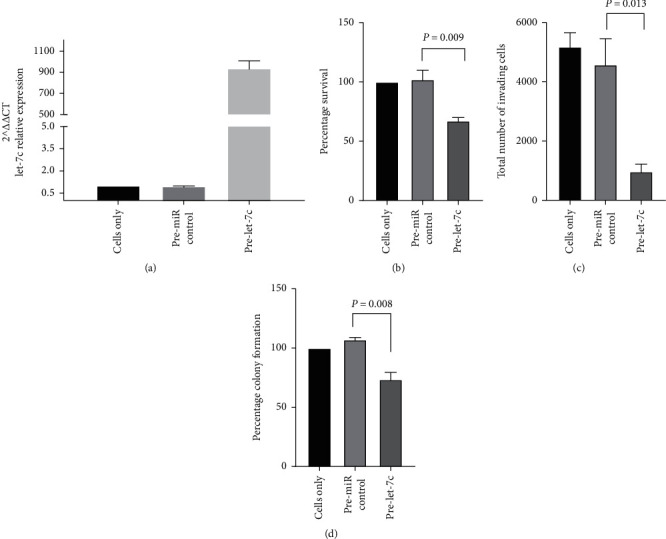
The biological function of let-7c in the progression and invasion was investigated by transiently inducing (a) let-7c into Panc-1 cell line; (b) restoration of miRNA let-7c in Panc-1 cells significantly reduced proliferation; and (c) invasion and (d) colony formation efficiency compared with pre-miR control.

The biological function of let-7c in the progression and invasion of pancreatic cancer was investigated by transiently inducing mimic pre-miR-let-7c into the Panc-1 cell line. Panc-1 was used as this cell line displayed the highest invasion of all the cell lines; therefore, inhibition of invasion would be more quantifiable in this cell line. We confirmed using qRT-PCR that the induced let-7c expression in the pre-let-7c transfected Panc-1 cells was significantly higher compared with the negative control (pre-miR ctrl) (*P* value <0.0001) and in the untreated cells after 72 h (Figure 3(a)). Transient overexpression of let-7c in Panc-1 cells significantly decreased proliferation (*P* value = 0.009) (Figure 3(b)), reduced invasive abilities by 4 (*P* value = 0.013) (Figure 3(c)), and prevented the formation of colonies by 33 ± 4.6% compared with pre-miR mimic control (*P* value = 0.008) (Figure 3(d)).

### 3.4. Predictive Target Genes of let-7c Using Differential mRNA Expression and *In Silico* miRNA-Targeted Gene Prediction Analysis

Gene expression microarray analysis was performed using the same high-invasive Clone #3 and low-invasive Clone #8 subclonal cell lines with the aim of identifying differentially expressed genes that might be target gene candidates for the differentially expressed miRNAs. The Affymetrix GeneChip analysis showed that 1074 genes (601 genes upregulated and 473 genes downregulated) were differentially expressed (*P* value <0.05 and fold change = ±2) between high-invasive and low-invasive cell lines (Supplementary Figures 1 and 2). The up/downregulated gene lists were compared with the lists of predictive (algorithm based)/experimental target genes of let-7c (Table 1). Four putative target genes of let-7c were predicted using the anticorrelated gene expression between high- versus low-invasive cell lines and by applying the five databases as described in Materials and Methods.

### 3.5. Expression of *SOX13* in Pancreatic Cancer

**Figure 4:**
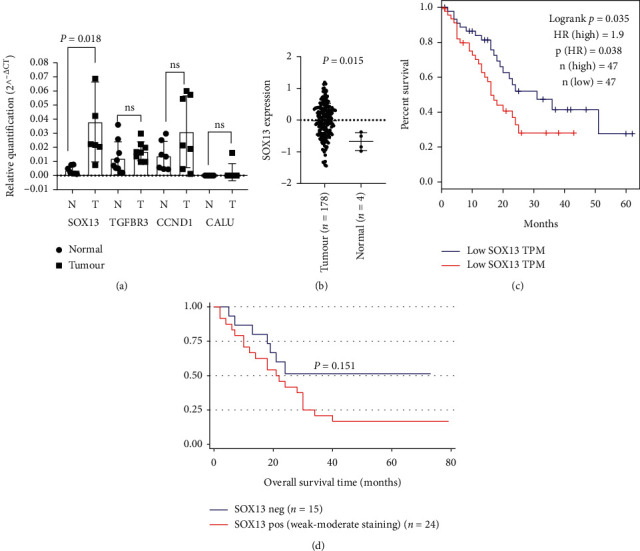
(a) Expression of miR-let-7c potential gene target *SOX13* in adjacent normal pancreas and PDAC tumour tissue. (b) *SOX13* mRNA significantly higher in TCGA pancreatic tumour compared with normal tissue. (c) Kaplan–Meier overall survival of TCGA pancreatic cancer patient's cut-off into low vs. high *SOX13* mRNA expression. (d) Kaplan–Meier overall survival of independent cohort of pancreatic cancer patients dichotomized into negative vs. positive *SOX13* protein expression by IHC.

**Figure 5:**
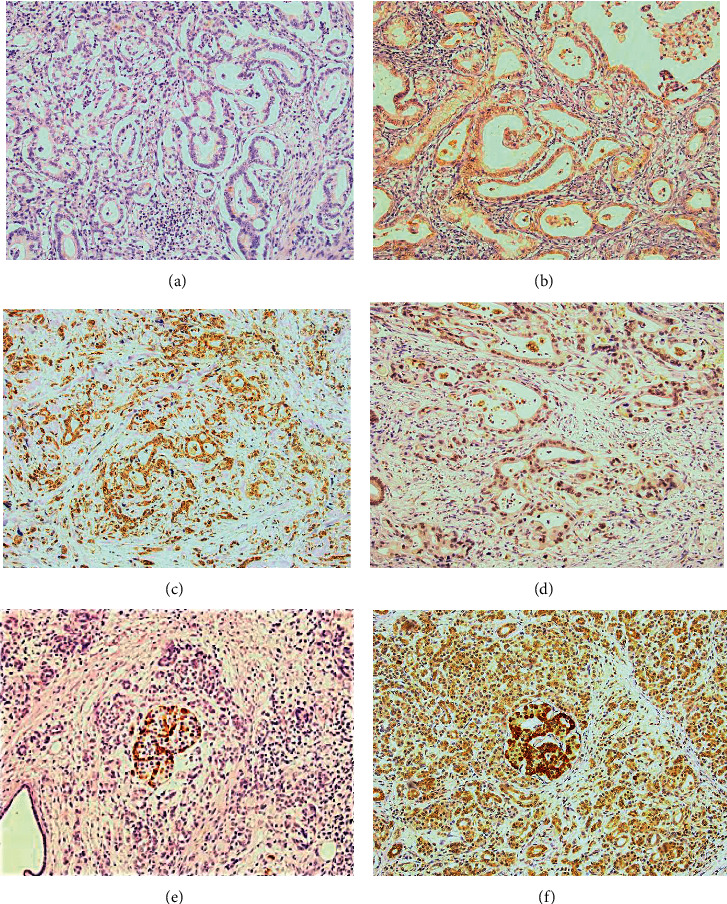
Immunohistochemistry of independent pancreatic cancer cohort stained for *SOX13*. Representative images show (a) negative, (b) weak positivity, (c) moderate positivity staining of cytoplasm, (d) moderate nuclear staining, (e) strong *SOX13* expression in benign islet cells with negative acinar staining, and (f) strong *SOX13* expression in benign islet cells with positive acinar staining.

All four putative target genes were assessed for their expression between PDAC tumour and adjacent normal tissue. *SOX13* was the only gene that displayed significant differential expression between adjacent normal and tumour pancreas tissue (*P* value = 0.018) (Figure 4(a)). The expression of *SOX13* in PDAC tumour compared with normal pancreas was then investigated in the TCGA PDAC data set; although only a small number of normal samples (*n* = 4) *SOX13* mRNA was significantly higher in tumour specimens compared with normal (*P* value = 0.015) (Figure 4(b)). Kaplan–Meier overall survival analysis of TCGA and GTEx PDAC data sets (GEPIA) [24] revealed a poor prognosis for patients with high *SOX13* expression (cut off 74% vs. 26%) (HR 1.9; *P* value = 0.038, log rank) (Figure 4(c)). Furthermore, we performed IHC on 39 PDAC FFPE samples and correlated *SOX13* protein expression with overall survival and observed poorer survival among patients expressing *SOX13* than those with no expression; however, this association was nonsignificant (HR 1.86, 95% Confidence Interval 0.78 to 4.42; *P* value = 0.151) (Figure 4(d)). IHC staining found that 62% of cases positively expressed *SOX13* protein with weak or moderate cytoplasmic/nuclear staining in pancreatic cancer lesions (Figures 5(a)–5(d)). Positive staining was combined as no *SOX13* was observed in normal pancreas ducts. Strong *SOX13* expression was also identified in islets cells both with low and high acinar cell staining (Figures 5(e) and 5(f)).

### 3.6. Role of *SOX13* in Invasion and Colony Formation in PDAC Cells

**Figure 6:**
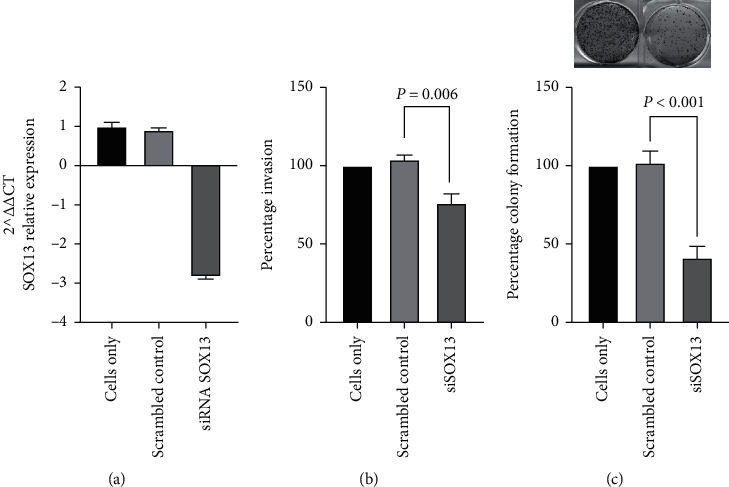
(a) Confirmation of knockdown of *SOX13* by siRNA by qRT-PCR. SiRNA inhibition of *SOX13* (siSOX13) significantly reduced (b) invasion and (c) colony formation efficiency in Panc-1 cell line (*n* = 3).

As *SOX13* showed differential expression between PDAC tumour and normal pancreas tissue, we decided to investigate its role in PDAC invasion and colony formation. Transient *SOX13* knockdown studies were performed with the highly invasive Panc-1 cell line.  Figure 6 illustrates a confirmed downregulation of *SOX13* by siRNA in Panc-1 cells. *SOX13* knockdown by siRNA resulted in significant decreased invasion (*P* value = 0.006) and reduced the colony formation ability of Panc-1 cells by 40% (*P* value = 0.0001) compared with scrambled siRNA control Panc-1 cells (Figures 6(b) and 6(c)).

## 4. Discussion

Pancreatic cancer represents a major clinical challenge, as it is an aggressive tumour, which invades and metastasizes quickly. Understanding the mechanisms underlying these events is critical to develop new biomarkers and therapeutics. miRNAs bind to the 3′UTR of their target genes playing decisive roles in proliferation, apoptosis, invasion, and metastasis in cancer. In this study, we identified significant differentially expressed miRNAs between high- and low-invasive subpopulations of the pancreatic cancer cell line MiaPaCa-2. Expression of the top upregulated (miR-135b) and downregulated (let-7c) miRNAs positively and inversely correlated with the invasive abilities of a panel of pancreatic cancer cell lines and showed differential expression between adjacent normal and pancreatic cancer tissue. Restoration of let-7c in the PDAC cell line Panc-1 decreased proliferation, invasion, and colony formation abilities. Let-7c is a tumour suppressor miRNA downregulated in many cancers, and its expression suppresses proliferation, inhibits migration and invasion, induces cell apoptosis, and disrupts the cell cycle [25, 26]. In pancreatic cancer, the let-7 family is an established tumour suppressor with downregulation of let-7g associated with shorter overall survival [27, 28]. Induced expression of let-7a inhibits *in vitro* proliferation due to targeting of the *KRAS*-dependent pathway [29]. Patel et al. found that re-expression of let-7c in poorly differentiated PDAC cell lines reduced phosphorylation/activation of *STAT3* and its downstream signalling events and reduced the growth and migration of PDAC cells [14]. miRNAs such as let-7c have the ability to suppress multiple target genes. Enhanced expression of tumour-suppressive miRNAs and suppression of oncogenic target genes may offer more effective treatment strategies. We integrated miRNA-mRNA gene expression correlation analysis using *in silico* computational databases to identify novel gene targets. We found that *SOX13* (SRY-related high mobility group box transcription factor 13) remained significantly upregulated in PDAC tumours compared with normal pancreas tissue in the pancreatic cancer cohort of TCGA [30] and in an independent data set and was significantly associated with poorer survival. *SOX13* is upregulated in tumours such as renal clear cell carcinoma [31] and colorectal cancer [32]. *SOX13* is a target gene of let-7i in colorectal cancer [32] and miR-138-5p circ_002136-mediated glioma angiogenesis [33]. Recently, Du et al. observed that overexpression of *SOX13* promoted migration, invasion, and metastasis via activation of c-MET and SNAI2 in colorectal cancer [34]. In agreement, our study found that knockdown of *SOX13* by siRNA reduced the invasive and colony formation abilities of the high-invasive Panc-1 PDAC cell line, indicating a role for *SOX13* for the first time in invasion and progression of PDAC.

In order to address the limitations of this study, the precise mechanism of let-7c/*SOX13* axis by luciferase reporter assays in PDAC must be elucidated. Additionally, the localization of *SOX13* staining in islet cells should be explored further, particularly, as *SOX13* is a type-1 diabetes autoantigen (also known as islet cell antibody 12) [35]. Zhang et al. found that 6% of 33 Chinese type-1B diabetic patients were found positive for SOX13-Ab, indicating islet autoimmunity [36]. Challenges may exist to unravel the complex role of *SOX13*, diabetes, and cancer as *SOX13* has been shown to be a nonspecific marker of pancreas tissue damage associated with chronic hyperglycaemia [37]. Utilizing GeneCards [38] Genes Like Me analysis tool highlighted shared descriptors between *SOX13* and top genes in the Maturity Onset Diabetes of the Young (MODY) pathway such as *HNF4G*, *HNF1B*, *HNF1A*, *INS*, *PDX1*, *NEUROD1*, and *GCK*. Our previous research in pathway analysis of genome-wide association study (GWAS) data on PDAC found that the top single nucleotide polymorphisms (SNPs) were associated with genes in the MODY pathway, suggesting that this pathway may be biologically relevant for risk of PDAC [39]. However, the association of *SOX13* in MODY or PDAC has not been fully elucidated, and its function may be resolved to its role as a transcription factor, effective regulator of embryonic development, stem cell maintenance, tissue homeostasis, and multiple cancer development [40].

In conclusion, downregulation of let-7c and overexpression of *SOX13* play a role in pancreatic cancer. In order to develop new treatment approaches for pancreatic cancer, the therapeutic value of targeting *SOX13* to reduce pancreatic cancer invasion should be further validated by independent cohorts and prospective trials using key agents such as crizotinib previously shown to block key *SOX13*-related pathways [34] and additional exploration of the regulatory network of *SOX13* to elucidate additional molecular targeted agents for *SOX13*-overexpressing PDAC patients.

## Figures and Tables

**Table 1:** Putative target genes of let-7c using anticorrelation mRNA gene signature.

miRNA	mRNA microarrays			*In silico* algorithm and experimental databases				
TLDA miR array	Gene symbol	High vs. low invasion	*P* value	miRWalk	TarBase	miRDB	PicTar	Target scan
		Fold change						
let-7c	CCND1	2.01	0.005	—	X	—	X	X
	CALU	2.10	0.004	X	X	—	X	X
	*SOX13*	2.70	0.046	X	—	X	X	X
	TGFBR3	9.96	0.009	X	X	X	—	X

## Data Availability

The data sets used during the present study are available from the corresponding author on reasonable request.

## Supplementary Materials

Supplementary Materials Supplementary Figure 1: differential mRNA gene expression using Affymetrix human genome u133 plus 2 microarray. (A) Hierarchical and (B) heat map of invasive Clone#3 and noninvasive Clone#8 cells show 1074 genes differentially regulated (601 upregulated and 473 downregulated) at a fold difference of ±2 and *P* value <0.05. Supplementary Figure 2: schematic layout of experimental design and experimental processes.

## References

[1] Bray F., Ferlay J., Soerjomataram I., Siegel R. L., Torre L. A., Jemal A. (2018). Global cancer statistics 2018: GLOBOCAN estimates of incidence and mortality worldwide for 36 cancers in 185 countries. *CA: A Cancer Journal for Clinicians*.

[2] Ferlay J., Partensky C., Bray F. (2016). More deaths from pancreatic cancer than breast cancer in the EU by 2017. *Acta Oncologica*.

[3] Rahib L., Smith B. D., Aizenberg R., Rosenzweig A. B., Fleshman J. M., Matrisian L. M. (2014). Projecting cancer incidence and deaths to 2030: the unexpected burden of thyroid, liver, and pancreas cancers in the United States. *Cancer Research*.

[4] Oettle H., Neuhaus P., Hochhaus A. (2013). Adjuvant chemotherapy with gemcitabine and long-term outcomes among patients with resected pancreatic cancer. *Journal of the American Medical Association*.

[5] Perini M. V., Montagnini A. L., Jukemura J. (2008). Clinical and pathologic prognostic factors for curative resection for pancreatic cancer. *HPB: The Official Journal of the International Hepato Pancreato Biliary Association.*.

[6] Lin S., Gregory R. I. (2015). MicroRNA biogenesis pathways in cancer. *Nature Reviews Cancer*.

[7] Zhao G., Wang B., Liu Y. (2013). miRNA-141, downregulated in pancreatic cancer, inhibits cell proliferation and invasion by directly targeting MAP4K4. *Molecular Cancer Therapeutics*.

[8] Wang C., Liu P., Wu H. (2016). MicroRNA-323-3p inhibits cell invasion and metastasis in pancreatic ductal adenocarcinoma via direct suppression of SMAD2 and SMAD3. *Oncotarget*.

[9] Zhu G., Zhou L., Liu H., Shan Y., Zhang X. (2018). MicroRNA-224 promotes pancreatic cancer cell proliferation and migration by targeting the TXNIP-mediated HIF1*α* pathway. *Cellular Physiology and Biochemistry*.

[10] Zhang Y., Su Y., Zhao Y., Lv G., Luo Y. (2017). MicroRNA-720 inhibits pancreatic cancer cell proliferation and invasion by directly targeting cyclin D1. *Molecular Medicine Reports*.

[11] Pasquinelli A. E., Reinhart B. J., Slack F. (2000). Conservation of the sequence and temporal expression of let-7 heterochronic regulatory RNA. *Nature*.

[12] Su J.-L., Chen P.-S., Johansson G., Kuo M.-L. (2012). *MicroRNA: Function and Regulation of Let-7 Family MicroRNAs*.

[13] Zhao L., Chen X., Cao Y. (2011). New role of microRNA: carcinogenesis and clinical application in cancer. *Acta Biochimica et Biophysica Sinica*.

[14] Patel K., Kollory A., Takashima A., Sarkar S., Faller D. V., Ghosh S. K. (2014). MicroRNA let-7 downregulates STAT3 phosphorylation in pancreatic cancer cells by increasing SOCS3 expression. *Cancer Letters*.

[15] Johnson S. M., Grosshans H., Shingara J. (2005). RAS is regulated by the let-7 MicroRNA family. *Cell*.

[16] Walsh N., Clynes M., Crown J., O’Donovan N. (2009). Alterations in integrin expression modulates invasion of pancreatic cancer cells. *Journal of Experimental & Clinical Cancer Research*.

[17] Walsh N., Larkin A., Swan N. (2011). RNAi knockdown of Hop (Hsp70/Hsp90 organising protein) decreases invasion via MMP-2 down regulation. *Cancer Letters*.

[18] Niyazi M., Niyazi I., Belka C. (2007). Counting colonies of clonogenic assays by using densitometric software. *Radiation Oncology*.

[19] Krek A., Grün D., Poy M. N. (2005). Combinatorial microRNA target predictions. *Nature Genetics*.

[20] Agarwal V., Bell G. W., Nam J.-W., Bartel D. P. (2015). Predicting effective microRNA target sites in mammalian mRNAs. *Elife*.

[21] Liu W., Wang X. (2019). Prediction of functional microRNA targets by integrative modeling of microRNA binding and target expression data. *Genome Biology*.

[22] Sticht C., De La Torre C., Parveen A., Gretz N. (2018). miRWalk: an online resource for prediction of microRNA binding sites. *PLoS One*.

[23] Karagkouni D., Paraskevopoulou M. D., Chatzopoulos S. (2018). DIANA-TarBase v8: a decade-long collection of experimentally supported miRNA-gene interactions. *Nucleic Acids Research*.

[24] Tang Z., Li C., Kang B., Gao G., Li C., Zhang Z. (2017). GEPIA: a web server for cancer and normal gene expression profiling and interactive analyses. *Nucleic Acids Research*.

[25] Fu X., Mao X., Wang Y., Ding X., Li Y. (2017). Let-7c-5p inhibits cell proliferation and induces cell apoptosis by targeting ERCC6 in breast cancer. *Oncology Reports*.

[26] Nadiminty N., Tummala R., Lou W (2012). MicroRNA let-7c is downregulated in prostate cancer and suppresses prostate cancer growth. *PLoS One*.

[27] Jiao L. R., Frampton A. E., Jacob J. (2012). MicroRNAs targeting oncogenes are down-regulated in pancreatic malignant transformation from benign tumors. *PLoS One*.

[28] Schultz N. A., Andersen K. K., Roslind A., Willenbrock H., Wøjdemann M., Johansen J. S. (2012). Prognostic MicroRNAs in cancer tissue from patients operated for pancreatic cancer-five MicroRNAs in a prognostic index. *World Journal of Surgery*.

[29] Rô me Torrisani J., Bournet B., Chalret du Rieu M. (2009). Let-7 MicroRNA transfer in pancreatic cancer-derived cells inhibits in vitro cell proliferation but fails to alter tumor progression. *Human Gene Therapy*.

[30] Cancer Genome Atlas Research Network, Raphael B. J., Hruban R. H. (2017). Integrated genomic characterization of pancreatic ductal adenocarcinoma. *Cancer Cell*.

[31] Gu W., Wang B., Wan F. (2018). SOX2 and SOX12 are predictive of prognosis in patients with clear cell renal cell carcinoma. *Oncology Letters*.

[32] Zhang P., Ma Y., Wang F. (2012). Comprehensive gene and microRNA expression profiling reveals the crucial role of hsa-let-7i and its target genes in colorectal cancer metastasis. *Molecular Biology Reports*.

[33] He Z., Ruan X., Liu X. (2019). FUS/circ_002136/miR-138-5p/*SOX13* feedback loop regulates angiogenesis in Glioma. *Journal of Experimental & Clinical Cancer Research*.

[34] Du F., Li X., Feng W. (2020). *SOX13* promotes colorectal cancer metastasis by transactivating SNAI2 and c-MET. *Oncogene*.

[35] Kasimiotis H., Myers M. A., Argentaro A. (2000). Sex-determining region Y-related protein *SOX13* is a diabetes autoantigen expressed in pancreatic islets. *Diabetes*.

[36] Zhang D., Zhou Z., Li L. (2006). Islet autoimmunity and genetic mutations in Chinese subjects initially thought to have Type 1B diabetes. *Diabetic Medicine*.

[37] Davis T. M. E., Mehta Z., Mackay I. R. (2003). Autoantibodies to the islet cell antigen SOX-13 are associated with duration but not type of diabetes. *Diabetic Medicine*.

[38] Stelzer G., Rosen N., Plaschkes I. (2016). The GeneCards suite: from gene data mining to disease genome sequence analyses. *Current Protocols in Bioinformatics*.

[39] Walsh N., Zhang H., Hyland P. L. (2018). Agnostic pathway/gene set analysis of genome-wide association data identifies associations for pancreatic cancer. *Journal of the National Cancer Institute*.

[40] Roose J., Korver W., De Boer R., Kuipers J., Hurenkamp J., Clevers H. (1999). TheSox-13Gene: structure, promoter characterization, and chromosomal localization. *Genomics*.

