# Supplementary figures and images for: Genomic Profiling and Functional Analysis of let-7c miRNA-mRNA Interactions Identify SOX13 to Be Involved in Invasion and Progression of Pancreatic Cancer
Source: J Oncol. 2020 Dec 24;2020:2951921. doi: 10.1155/2020/2951921 (PMC7775161; doi:10.1155/2020/2951921)

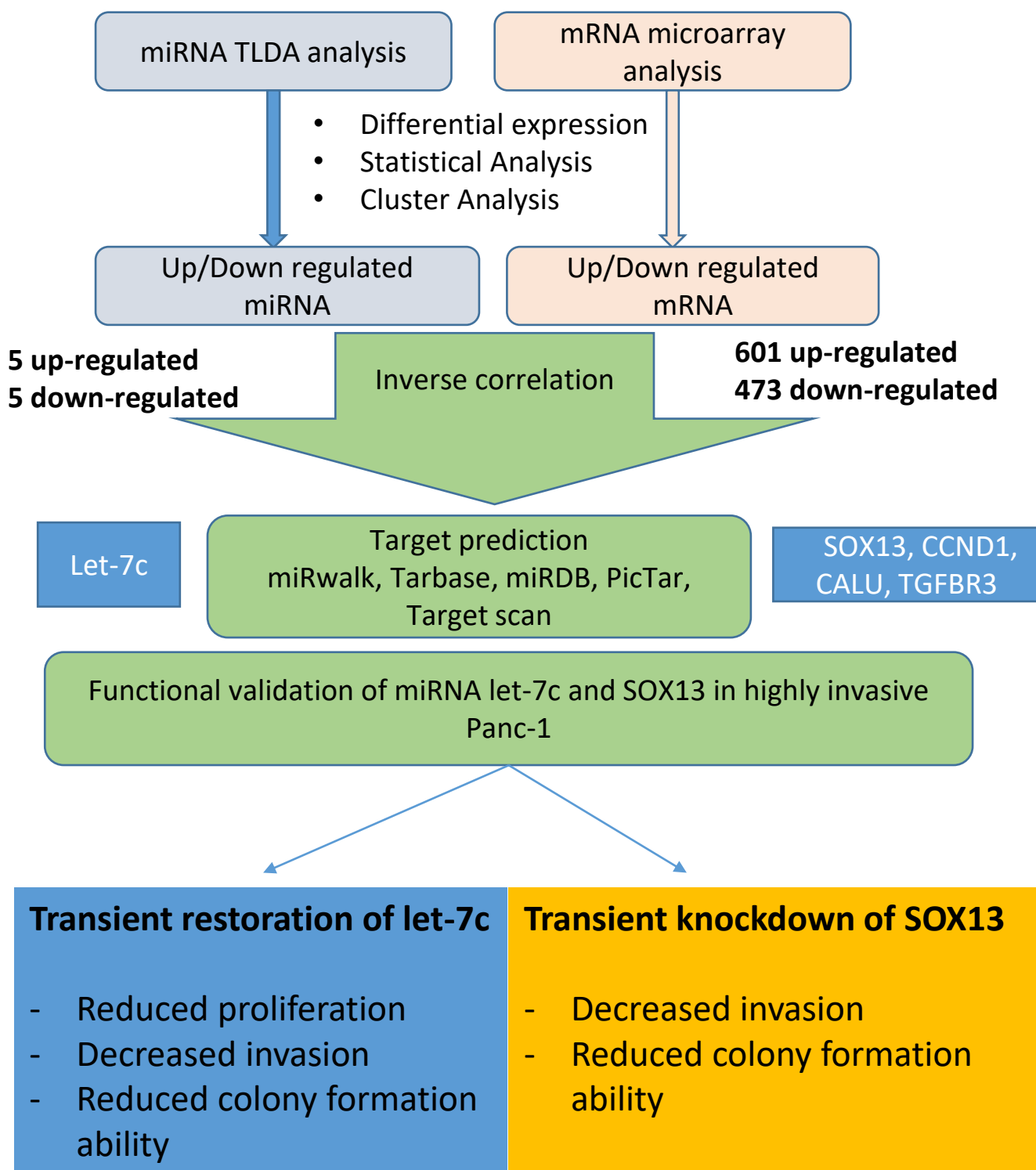

Supplement: Supplementary Materials — Supplementary Figure 1: differential mRNA gene expression using Affymetrix human genome u133 plus 2 microarray. (A) Hierarchical and (B) heat map of invasive Clone#3 and noninvasive Clone#8 cells show 1074 genes differentially regulated (601 upregulated and 473 downregulated) at a fold difference of ±2 and P value <0.05. Supplementary Figure 2: schematic layout of experimental design and experimental processes. [file 2951921.f1.zip › 2951921.f1/Supplementray figure 2.pdf]

**A.**

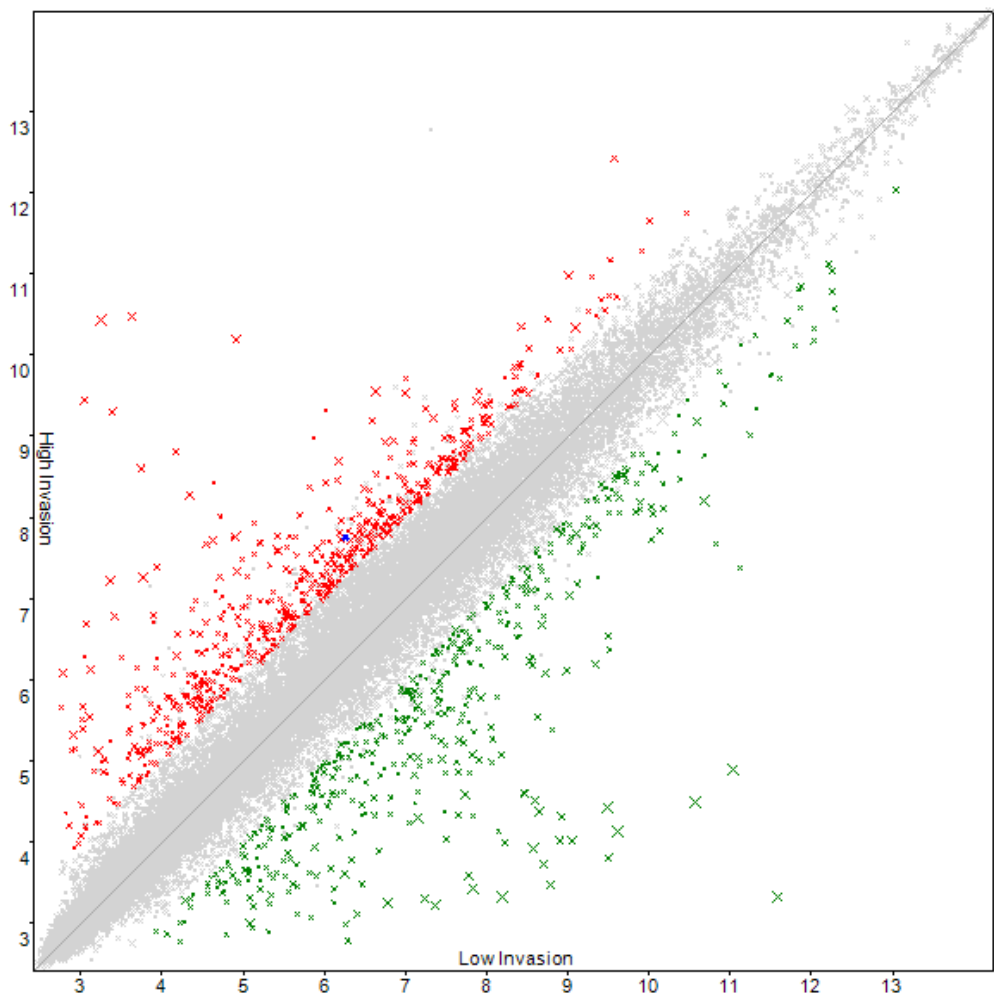

**B.**

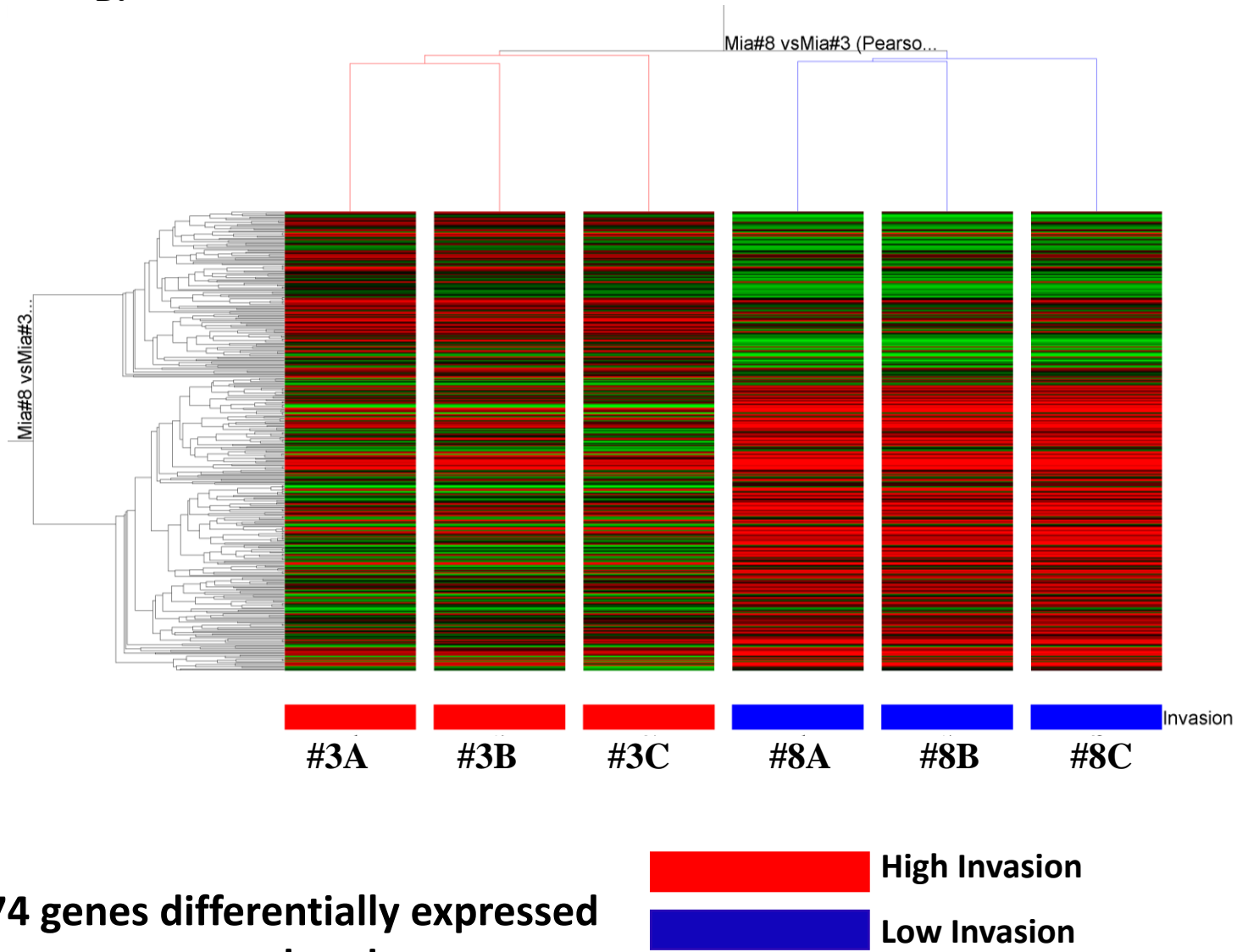

**1074 genes differentially expressed**  
**601 genes up-regulated**  
**473 genes down-regulated**

Supplement: Supplementary Materials — Supplementary Figure 1: differential mRNA gene expression using Affymetrix human genome u133 plus 2 microarray. (A) Hierarchical and (B) heat map of invasive Clone#3 and noninvasive Clone#8 cells show 1074 genes differentially regulated (601 upregulated and 473 downregulated) at a fold difference of ±2 and P value <0.05. Supplementary Figure 2: schematic layout of experimental design and experimental processes. [file 2951921.f1.zip › 2951921.f1/Supplemetary figure 1.pdf]
